# Antimicrobial potential of the Ethiopian *Thymus schimperi* essential oil in comparison with others against certain fungal and bacterial species

**DOI:** 10.1186/s12906-015-0784-3

**Published:** 2015-07-31

**Authors:** Mohammed Nasir, Ketema Tafess, Dawit Abate

**Affiliations:** Department of Medical Laboratory Sciences, College of Health Sciences, Arsi University, Asella, Ethiopia; Department of Microbial, Cellular and Molecular Biology, College of Natural Sciences, Addis Ababa University, Addis Ababa, Ethiopia

**Keywords:** *Thymus schimperi*, *Cinnamomum zeylanicum*, *Tricophyton*, *Escherichia coli*

## Abstract

**Background:**

To evaluate the in vitro activities of Ethiopian *Thymus schimperi* with other three hydro distilled essential oils against Dermatophytes (*Tricophyton spp. and Microsporum spp*.) and other pathogenic micro organisms.

**Methods:**

The studies were carried out using Agar disk diffusion method for screening the most effective essential oils and Agar dilution to determine Minimum Inhibitory Concentration (MIC) of the essential oils.

**Results:**

Essential oils of *T. schimperi* and *Cinnamomum zeylanicum* were highly active against tested organisms. The MIC were in the range of 0.08 μl/ml to 0.31 μl/ml for *T. schimperi,* 0.31 μl/ml to 0.16 μl/ml for *C. zeylanicum*, 2.5 μl/ml to1.25 μl/ml for *Citrus limon* and 5 μl/ml to 2.5 μl/ml for *Eucalyptus camaldulensis* against *Tricophyton spp*. and *Microsporum spp. T. schimperi* and *C. zeylanicum* oils also showed antimicrobial effect against *Candida albicans*, *Aspegilus niger*, *Rhodotorula rubra, Escherichia coli*, *Shigella spp*., *Bacillus spp*. and *Streptococci.*

**Conclusions:**

The Ethiopian *T. schimperi* oil had pronounced antifungal and antibacterial activities against all the tested microbes. Therefore, it is required further investigation in order to identify the active compounds and their clinical applications for treatment of tested organisms.

## Background

Antimicrobial properties have been reported more frequently in a wide range of plant extracts and essential oils and natural products in an attempt to discover new chemical classes of antifungal and antibacterial drugs that could resolve strains expressing resistance to the available antifungal and antibacterial drugs [1, 2].

Essential oils are volatile, natural, complex compounds characterized by a strong odor and are formed by aromatic plants as secondary metabolites. They are usually obtained by steam or hydro-distillation [3, 4]. At present, approximately 3000 essential oils are known, 300 of which are commercially important especially for the pharmaceutical, agronomic, food, sanitary, cosmetic and perfume industries [5].

*T. pulegioides* essential oil has potential as a topical antifungal agent against Dermatophytes, *Aspergilus*, and *Candida* [6]. Other species of the genus *Thymus*, such as *T. zygis* and *T. vulgaris*, with high amounts of phenols, also show a broad spectrum of activity against a variety of pathogenic yeasts and filamentous fungi, including fungi with decreased susceptibility to fluconazole [6].

The *Thymus* species in Ethiopia are restricted to afromontane and afro-alpine regions and are represented by the two endemic species, *T. schimperi* Ronninger and *T. serrulatus* Hochst [7]. They have a crowded inflorescence with pink corollas and have ovate to elliptic leaves with entire margins [8]. They are extensively used by local people as food preservatives, cure for various ailments and food flavoring and seasonings [8]. However, the antimicrobial activity of *T. schimperi* essential oil has not been widely investigated. Therefore, in order to fill this gap of information, essential oil of this plant is to be investigated for its potential in comparison with other essential oils in the control of *Trichophyton* spp., *Microsporum spp., C. albicans*, *A. niger, R. rubra* and different bacteria strains (*E. coli, Bacillus spp., Shigella spp. and Streptococci*) grown in vitro using Agar disk diffusion and Agar dilution method.

## Methods

### Plant material and preparation of essential oils

Four various types of herbs and spice plants were collected around Addis Ababa: *T. schimperi* (leaf with aerial parts), *C. zeylanicum* (bark), *C. limon* (peel), and *E. camaldulensis* (leaf) and identified by the national herbarium of Ethiopia. The plants were brought to the laboratory and thoroughly washed in running tap water to remove debris and dust particles and then rinsed in distilled water. The collected plant materials were dried at room temperature and ground into a semi powder using a grinder.

Each semi powdered plant materials were hydro distilled by a Clevenger apparatus at 70 °C – 80 °C for 3-5 hrs (approximately four times water (w/v) in volume than the plant material). The extracted fractions of plant parts exhibited two distinct layers an upper oily layer and the lower aqueous layer. Both the layers were separated and oils were collected in clear vials, after removing water traces using anhydrous sodium sulfate, and they were stored at 4 °C until needed.

### Microorganisms

*Tricophyton spp*., *Microsporum spp.* and *C. albicans* were obtained from Ethiopian Health and Nutrition Institute (EHNRI) while local isolates of *A. niger* and *R. rubra* and four bacteria strains (*E. coli, Bacillus spp., Shigella spp. and Streptococci)* were obtained from Addis Ababa University, Mycology and Bacteriology laboratory. Sabouraud Dextrose Agar was used as growing medium for *Tricophyton spp*., *Microsporum spp.* and *C. albicans* while Potato dextrose agar (PDA; Himedia Ltd., India) for *A. niger* and *R. rubra*. The bacteria were grown in nutrient broth and Mueller – Hinton agar. Periodic transfers were done to keep the micro organism viable.

## The antimicrobial assays

### Disk diffusion assay

The antimicrobial activity test of the essential oils were carried out by the agar diffusion method, which is normally used as a preliminary screening of efficient essential oils [9] following the procedure approved by NCCLS (2002) with little modification. The agar plate was prepared for each organism as follows: a suspension of the organism (20 μl and 0.1 ml of inoculums suspension for fungi and bacteria respectively) of the standardized inoculums was mixed with 20 ml of sterile Sabouraud Dextrose ® Agar for *Tricophyton spp*., *Microsporum spp.* and *C. albicans,* PDA for *A. niger* and *R. rubra* and Nutrient agar for bacteria then poured into sterilized Petri dishes and set aside. After solidified, sterile filter paper disc (Whatman no.1, 6 mm in diameter) impregnated with 5 μl of each essential oil were placed over the middle of the plates already seeded test organism. Sterile distilled water but not oil was added on the discs to provide negative control and as a positive control Griseofulvin (0.03 mg/ml) and Chloramphenicol (0.01 mg/ml) for fungi and bacteria respectively were provided by Addis Ababa University, Department of Microbiology; Mycology Laboratory. The plates were incubated at 37 °C for 10 days for Dermatophytes and plates with *Candida, Aspergillus and Rhodotorula* for 48 h and plates with bacteria were incubated for 24 h. Each test was done in triplicates. The antimicrobial activity was evaluated by measuring the diameter of the zone of inhibition (including diameter of the disk) [9].

### Determination of minimum inhibitory concentration (MIC)

The essential oils that previously showed antimicrobial activity were screened for determination of MIC by Agar dilution method [10]. The agar dilution method followed that approved by the National Committee for clinical laboratory standards NCCLS (2000) with little modification. Two fold serial dilutions in DMSO ranging from 0.019 to 20 μ/ml were tested for each essential oil. The prepared plates were incubated and evaluated for the presence or absence of microbial growth. The MIC was determined as the lowest concentration of oil inhibiting the visible growth of each organism on the plates [10].

### Statistical analysis

All the measurements were replicated three times for each assay and the results are presented as mean standard deviation. The statistical analysis was performed by one-way analysis of variance (ANOVA) and P values < 0.05 were considered as significant.

## Results

Preliminary screening of the antifungal activity *in vitro* of the four essential oils was studied against fungal pathogens using the filter paper disc agar diffusion technique as shown in (Table 1). All the oils tested exhibited different degrees of antifungal activity against *Tricophyton spp, Microsporum* spp. and other tested fungi. The maximum antimycotic activity was shown by *T. schimperi* followed by *C. zeylanicum. T. schimperi* has shown the greatest inhibition zone diameter of 88.66 mm against *R. rubra* and 73.33 mm against Clinical isolates of *Ttricophyton spp*. and *Microsporum spp.* The least inhibition zone exhibited by *T. schimperi was* 35.66 mm against *C. albicans*. Similarly, *C. zeylanicum* inhibited with the range of 31.33 mm to 62.66 mm against all the tested fungi and considered as a high result next to *T. schimperi.* The oils of *C. limon* (lemon) exhibited moderate activity and the oils of *E. camaldulensis* showed comparatively low activity against all the tested organisms. Control disks with distilled water showed no inhibition in a preliminary test. The difference between the essential oils was statistically significant as proofed from the results of one-way ANOVA (Table 1).

**Table 1:** Antifungal activity of four essential oils against the tested fungi using disk diffusion assay. (Mean inhibition zone diameter including disk diameter)

Tested fungi	Inhibition zone diameter in mm (Mean ± SD)				
	Thymus	Cinnamon	Lemon	Eucalyptus	Grisiofulvin
	EO*	EO	EO	EO	
*Tricophyton* spp. (scalp isolate)	73.33 ± 2.081	61.00 ± 2.000	15.33 ± 0.577	5.66 ± 1.155	36.00 ± 1.000
*Tricophyton* spp. (nail isolate)	72.33 ± 2.08	62.66 ± .527	16.33 ± 0.577	6.66 ± 1.527	32.66 ± 1.577
*Microsporum spp.*	73.00 ± 1.000	62.00 ± 2.081	14.66 ± 0.577	3.66 ± 3.214	34.00 ± 2.000
*Candida albicans*	35.66 ± 1.527	31.33 ± 1.527	15.66 ± 0.577	0.00 ± 0.00	N.T+
*Aspergilus niger(Local isolates)*	62.66 ± 1.527	54.66 ± 2.081	15.33 ± 1.527	10.66 ± 0.57	N.T
*Rhodotorula rubra( Local isolate)*	88.66 ± 2.309	54.00 ± 1.000	42.00 ± 1.00	10.00 ± 2.00	N.T

+N.T: Not tested, *EO: Essential oil

Bacteria susceptibility to the essential oils also determined by the agar diffusion method and showed that *T. schimperi* oil possessed potential antibacterial activity against *E. coli, Bacillus spp., Shigella spp. and Streptococci.* The highest antibacterial activity of *T. schimperi* oil was 90 mm in *E.coli*, *Shigella spp*. and *Striptococci* and relatively least activity was recorded in *Bacillus sp*. measured 63 mm (Table 2). *C. zeylanicum* exhibited almost the highest activity against all the tested bacteria which measured in the range of 36 mm-43 mm as shown in (Table 2) next to *Thymus* oil. The oils of *C. limon* exhibited moderate activity and *E. camaldulensis* which has less or no effect with similar concentration (5 μl).

**Table 2:** Antibacterial activity of four essential oils against the tested bacteria using disk diffusion assay (Mean inhibition zone diameter including disk diameter)

Inhibition zone diameter including in mm (Mean ± SD)					
Tested					
Bacteria	Thymus	Cinnamon	Lemon	Eucalyptus	Chloramphenicol
	EO	EO	EO	EO	
*Escherichia*	90.00 ± 0.00	42.66 ± 1.527	26.66 ± 1.527	16.00 ± 2.645	43.33 ± 0.577
*coli*					
*Bacillus spp.*	63.00 ± 7.2	43.33 ± 1.527	25.66 ± 0.577	7.66 ± 5.89	34.00 ± 1.000
*Shigella spp.*	90.00 ± 0.00	36.66 ± 1.527	27.00 ± 1.000	0.00 ± 0.000	44.33 ± 0.577
*Streptococci*	90.00 ± 0.00	41.33 ± 2.081	20.33 ± 0.577	14.33 ± 0.577	42.66 ± 1.527

EO: Essential oil

As to the standard drugs used in the test, the inhibition zone for Grisofulvin was 36 mm, 34 mm and 32.66 mm for *Tricophyton spp.* (scalp isolate), *Microsporum spp and Tricophyton spp.* (nail isolate) respectively. The highest inhibition diameter recorded for Chloramphenicol was 44.33 mm against *Shigella spp.,* and the list was 34 mm against *Bacilus spp*. (Tables 1 and 2).

Evaluation of MIC showed that the *Thymus* and *Cinnamon* oils were active against all the tested isolates. *T. schimpri* oil showed the highest MIC values (0.08 μl/ml) against *Tricophyton spp*. (scalp isolate), *Microsporum spp.* and *R. rubra. Cinnamon* oil also has similar effect on *C. albicans*, *A. niger and R. rubra* (Table 3). The lowest concentration of the lemon essential oil at which the tested organisms (*Microsporum spp*. and *R. rubra)* were unable to grow was found to be ≥1.25 μl/ml. As can be noted from the Table 3, *Eucalyptus* essential oil inhibited growth of the *Tricophyton spp.* (scalp isolate) and *A. niger* at 2.5 μl/ml and for the rest of tested fungal strain at 5 μl/ml that were higher concentration when compared to the other oils.

**Table 3:** Minimum inhibitory concentrations (MIC) of the selected essential oils against the tested microorganisms

Test organisms and corresponding MIC (μl/ml)	*Thymus schimperi*	*Cinnamomum zeylanicum*	*Citrus limon*	*Eucalyptus camaldulensis*
*Tricophyton spp. *(nail isolate)	0.31	0.31	2.5	5
*Tricophyton spp. *(scalp isolate)	0.08	0.16	2.5	2. 5
*Microsporum spp.*	0.08	0.16	1.25	5
*Candida albicans*	0.16	0.08	2.5	5
*Aspegilus niger*	0.16	0.08	2.5	2. 5
*Rhodotorula*	0.08	0.08	1.25	5

MIC expressed in μl ml ^−1^ (v/v)

The data presented in Table 4 revealed variability in the inhibitory concentrations (MIC) of each extracts for tested bacteria. The essential oils from *Thymus* and *Cinnamon* showed activities in the range (concentration) from 0.31 μl/ml to 0.63 μl/ml and from 0.63 μl/ml to 1.25 μl/ml, respectively. Maximum activity was observed by *Thymus* oil against *Shigella* with MIC of 0.31 μl/ml. Lemmon oil exhibited modest antibacterial activity with 2.5 μl/ml MIC values against *E. coli*, *Shigella spp*., *Bacillus spp*. and *Streptcocci. Eucalyptus* oil was not as effective as others; it exhibited less antibacterial activity for all tested bacteria with MIC values of 5 μl/ml (Table 4).

**Table 4:** Minimum inhibitory concentrations (MIC) of the selected essential oils against the tested microorganisms

Test organisms and corresponding MIC (μl/ml)	*Thymus schimperi*	*Cinnamomum Zeylanicum*	*Citrus limon*	*Eucalyptus camaldulensis*
*E.coli*	0.63	1.25	2.5	5
*Shigella spp.*	0.31	1.25	2.5	5
*Bacillus spp.*	0.63	1.25	2.5	5
*Streptococci*	0.63	0.63	2.5	5

MIC expressed in μl ml ^−1^ (v/v)

## Discussion

In the preliminary screening of the essential oils, *T. schimperi* at 5 μl concentration caused higher inhibition zone with the range of 35.66 mm to 88.66 mm against tested fungi and 63 mm to 90 mm against bacteria. In similar study antimicrobial activity of seven essential oils from three *Thymus* species against *Salmonella enteritidis*, *Salmonella typhimurium, Eschericcha coli, Shigella flexneri* and *Listeria monocytogens* [11]. The result showed essential oil from *T. hyemalis* inhibited with the range of 19.6 mm to 45 mm. The existence of variations in the inhibition zone can be assumed that due to differences in the number of molecules and chemical type of molecules in the plant materials [12, 13].

In this study the essential oil extracted from *C. zeylanicum* bark demonstrated strong antifungal and antibacterial activity next to *T. schimperi.* The antifungal activity of *C. zeylanicum* exhibited 61 mm, 62.66 mm, 62 mm, 31.33 mm, 54.66 mm and 54 mm inhibition zone against *Tricophyton spp*. (scalp isolate), *Tricoophyton spp*. (nail isolate), *Microsporum spp.*, *C.albicans, A. niger* and *R.rubra* respectively. It also showed inhibition zone of 42.66 mm, 43.33 mm, 36.66 mm and 41.33 mm against *E. coli, Bacillus spp., Shigella spp*. and *Streptococci* respectively. The antimycotic activity of *C. zeylanicum* bark is due to presence of cinnamaldehyde [14].

In our work, the *C. limon* peel oil inhibited the tested fungi and bacteria in the range of 14.66 mm to 42 mm and 20.33 mm to 27 mm respectively and the antimicrobial effect is taken as a moderate activity when compared to *Thymus* and *Cinnamon* oils. However, the present study results were better than reported by (Gulay-Kirbasilar *et al*., [15]). This may be due to variations in climate and soil composition in which the plant growing [12, 13].

In this study, *E. camaldulensis* showed less inhibition zone against almost all the tested organisms. However, the result of *E. coli* and *Streptococci* were 16 mm and 14.3 mm respectively and taken as a moderate activity. In contrast to the present study, Babayi, *et al*., [16]) tested the efficacy of methanol extract of *E. camaldulensis* against *Salmonella typhi, Staphylococcus aureus* and *Bacillus subtilis and* reported a higher inhibition zone in a range of 15 mm-16 mm. In this case, the solvent extract of *E. camaldulensis* is better than the hydrodistiled essential oil against tested organism. According to Masotti *et al*., [13]) and Angioni *et al*., [12]), the extraction product can vary in quality, quantity and in composition according to the type of extraction method.

Some oils appeared more active with respect to Gram reaction, exerting a greater inhibitory activity against Gram positive bacteria [17]. However, the result obtained in the present study showed that the oil of *T. schimperi* did not show any selectivity towards the tested gram-positive and gram-negative bacteria. The substances extracted from thyme especially the phenolic components *thymol* and *carvacrol* showed antibacterial activity against gram-positive and gram- negative bacteria due to their effects on the bacterial membrane [7]. In this case, *T. schimperi* seems to better than other essential oils.

The MIC values of *T. schimperi* were with the range of 0.08 μl/ml to 0.031 μl/ml against

*Tricophyton s*pp. (scalp and nail isolate); *Microsporum spp.* and *R*. rubra. These results are in line with the work of Eugenia *et al*., [6] who had reported that *Thymys pulegioides* essential oil showed the lowest MIC value in the range of 0.16 μl/ml to 0.32 μ l/ml for Dermatophyte and 0.32 μl/ml to 0.64 μl/ml for *Candida*. This indicates that plants rich in a wide variety of secondary metabolites such as tannins, alkaloids, terpenoids which have been found *invitro* to have antimicrobial property [18]. *C. zeylanicum* oil also showed similar result with the same concentration on *Microsporum spp.*, *C. albicans*, *A.niger* and *R. rubra*.

The MIC of *C. limon* peel oil was 1.25 μl/ml against *R. rubra* and *Microsporum spp.* On the other hand, 2.5 μL/ml was recorded for the rest of the tested organisms. Therefore, *Rhodotorula* and *Microsporum* were more susceptible to *C. limon* peel oil than other tested organisms. However, the result obtained in limon oil was not comparable from the result of *T. schimperi* and *C. zeylanicum* which showed better antimicrobial activity for all tested organisms. This variation was due to the difference in plant type and part (organ) used in the experiment [12, 13]. In the present study, MIC of the essential oil of *E. camaldulensis* was in the range of 2.5 μl/ml to 5 μl/ml against all the tested organisms. The obtained results were in contrast to Morteza *et al*., [19]) who had recorded 1.95 μl/ml against *Staphylococcus aureus*. This variation may due to the difference in the tested organisms and the method used to assess antimicrobial activity (Janssen *et al*., [20]).

Our study has several limitations. There were no controls for *C. albicans, A. niger* and *R. rubra*, because the purpose was not comparison of the standard antimicrobial agent with plant materials. Moreover, all the tested organisms were not standards. However, the organisms were identified by cellular, cultural and biochemical characteristics. Finally, this study also did not include the chemical composition of plant materials due to financial shortage and lack of materials.

## Conclusion

The present study confirmed antimicrobial properties of essential oil from Ethiopian *T.* schimperi and showed significant growth inhibition for all the tested organisms. Therefore, these results will invite further study in the chemical components and the target cellular structure of the organisms.
